# “Brick‐Mortar‐Binder” Design toward Highly Elastic, Hydrophobic, and Flame‐Retardant Thermal Insulator

**DOI:** 10.1002/advs.202410938

**Published:** 2024-11-29

**Authors:** Shanying Sui, Huafeng Quan, Jingxing Wang, Yufang Lu, Yufan Yang, Yuhan Sheng, Zhifang Sun, Yi Zhang

**Affiliations:** ^1^ Hunan Provincial Key Laboratory of Micro & Nano Materials Interface Science College of Chemistry and Chemical Engineering Central South University Changsha 410083 P. R. China; ^2^ College of Materials Science and Engineering Hunan Province Key Laboratory for Advanced Carbon Materials and Applied Technology Hunan University Changsha 410082 P. R. China; ^3^ School of Materials Science and Engineering Xiangtan University Xiangtan Hunan 411105 P. R. China; ^4^ Key Laboratory of Oil and Gas Fine Chemicals of Ministry of Education College of Chemical Engineering Xinjiang University Urumqi 830017 P. R. China

**Keywords:** aerogels, elasticity, flame‐retardancy, hydrophobicity, thermal insulation

## Abstract

Advanced aerogels hold immense potential in thermal insulation. However, achieving high environmental adaptability aerogel insulators with elasticity, hydrophobicity, flame‐retardancy, and low temperature tolerance remains a significant challenge. Inspired by a “brick‐mortar‐binder” biomimetic texture, a layered double hydroxide/carboxylated cellulose nanofibers/Si–O–Si (LCS) hybrid aerogel is developed by bottom‐up freeze‐drying. Owing to the distinct building blocks and organized structure, as‐prepared LCS hybrid aerogel exhibits impressive mechanical elasticity, cycling stability at an extremely low temperature (‐196 °C), hydrophobicity, and flame‐retardancy (LOI = 44.6%, UL‐94: V‐0). Additionally, the incorporation of layered double hydroxide effectively improves the thermal insulation property (thermal conductivity = 0.0296 W·m^−1^·K^−1^). These distinctive features make the LCS hybrid aerogel highly promising for thermal management applications in extreme conditions, such as in pipelines for transporting liquid nitrogen and liquefied natural gas.

## Graphical abstract

Inspired by the “brick‐mortar‐binder” biomimetic texture, LCS hybrid aerogels are created through a directional freeze‐drying method. Owing to its distinct building blocks and organized structure, as‐prepared aerogel demonstrates outstanding elasticity, hydrophobicity, flame‐retardancy, and thermal insulation property. This hybrid aerogel is a promising thermal management material for extreme conditions, especially in low temperature and aqueous environments.

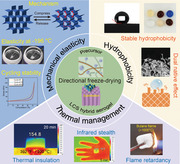

## 1 Introduction

Thermal energy plays a crucial role in various fields such as architecture, transportation, clothing, batteries, and aerospace.^[^ ^1^ ^]^ Efficient thermal energy management can not only reduce energy waste but also enhance the stability and lifespan of advanced equipment.^[^ ^2^ ^]^ Effective use of thermal management materials can significantly improve energy utilization efficiency. Cellulose aerogel has received considerable attention as a thermal insulation material due to its environment friendliness, biodegradability, machinability, and economical cost.^[^ ^3^ ^]^ However, achieving a balance between exceptional mechanical elasticity, hydrophobicity, flame‐retardancy, and thermal insulation in biomass aerogels remains a significant challenge. The construction of aerogels with these properties depends not only on the right composite materials employed but also on the innovative approaches to design a porous elastic network and microstructure.

It's well‐accepted that water absorption in porous aerogels can greatly reduce their thermal insulation effectiveness, given that water conducts heat much more efficiently than air.^[^ ^4^ ^]^ Additionally, water's erosive nature on hydrophilic materials can lead to structural collapse. Therefore, ensuring the hydrophobicity of thermal insulation aerogels is vital for their practical use. Cellulose aerogel formed by the physical cross‐linking of cellulose molecular chains often exhibits hydrophilicity, poor mechanical properties, and a low limiting oxygen index (LOI).^[^ ^5^ ^]^ Layered double hydroxides (LDHs), clay materials with positively‐charged laminated layers and interlayer anions, offer promising traits like flame‐retardancy and non‐volatility due to their large specific surface area and excellent thermodynamic properties (flame‐retardancy, halogen‐free, and non‐volatile traits).^[^ ^6^ ^]^ Hydrophobic Si–O–Si binder, generated through the hydrolysis and condensation of silane, is rich in –CH_3_ groups, making it an ideal hydrophobic agent. Integrating LDHs and Si–O–Si binder is thus crucial for achieving the hydrophobic, flame‐retardant, and thermal insulation properties needed in cellulose aerogel. Unfortunately, LDHs tend to aggregate through ab‐face stacking in solution due to their small size and high charge density.^[^ ^7^ ^]^ Previous reports have indicated that introducing hydrogen bonding, covalent bonding, or van der Waals forces between the LDHs and low‐dimensional nanomaterials or polymers carriers can help resolve the aggregation issue.^[^ ^8^ ^]^ Nevertheless, the interfacial connection between the carriers and LDHs may weaken during the complex preparation process of aerogels, thereby leading to heterogeneous composition. This heterogeneous composition limits the stress transfer within the LDHs‐based aerogel's 3D framework, resulting in insufficient mechanical robustness, which severely hinders the development and practical application.

Herein, we developed a hybrid LDH/CNF‐C/Si–O–Si aerogel (LCS) using a “brick‐mortar‐binder” one‐pot strategy. The aerogel comprises rigid LDH as the “brick,” flexible carboxylated cellulose nanofibers (CNF‐C) as the “mortar,” and hydrophobic Si–O–Si as the “binder.” In this strategy, the Si─O─Si bonded network, formed by the in situ mineralization of hydrolyzed silane coupling agents, effectively enhances interfacial bonding. It acts as a bridge to support the LDH nanoparticles on the CNF‐C carrier, addressing the issue of inadequate mechanical performance caused by stacking.

Apart from the building blocks, an effective structural design has been proven to be equally important for constructing functional aerogels. Scientists often view nature's intricately structured materials for inspiration in developing high‐performance materials. For instance, Yu et al. created a durable hybrid macrofiber inspired by spider silk.^[^ ^9^ ^]^ Zhang et al. produced a compressible aerogel inspired by biological bone.^[^ ^10^ ^]^ Their findings highlight the significant effectiveness of building hierarchical networks in the development of materials with exceptional mechanical properties and fatigue resistance. Leveraging these insights, this study employs directional freeze‐drying to meticulously manage ice crystal growth and adjust the aerogel's microstructure to meet specific performance objectives. As a result of this preparation strategy, our LCS hybrid aerogel exhibits many challenging requirements, including outstanding mechanical elasticity, robust stability, high hydrophobicity, exceptional flame‐retardancy, and remarkable thermal insulation property. The successful synthesis of this multifunctional LCS hybrid aerogel demonstrates its potential as a thermal management material capable of withstanding low temperature and aqueous environments.

## 2 Results and Discussion

### 2.1 Fabrication and Characterization

**Figure 1:**
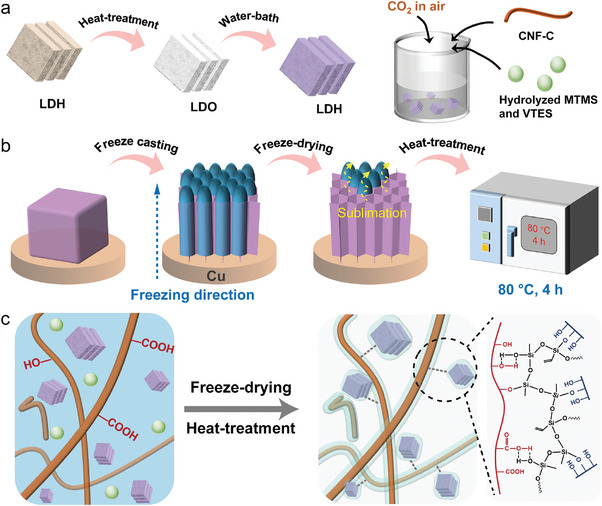
Preparation diagram of LCS hybrid aerogel. a) Preparation of LCS precursor solution. b) Fabrication of LCS hybrid aerogel. c) Processing principles.

**Figure** **1** illustrates the preparation procedure of LCS hybrid aerogel. Initially, hydrophobic LDH was calcined and then dispersed in deionized water at 80 °C. Due to the shape memory effect of LDH, the H_2_O and CO_2_ previously lost during calcination were reabsorbed from the surrounding water and air, thereby effectively regenerating the LDH.^[^ ^11^ ^]^ Afterward, hydrolyzed methyltrimethoxysilane (MTMS) and vinyltriethoxysilane (VTES), LDH, and CNF‐C were co‐mixed to construct a stable precursor suspension (Figure 1a), which was then placed on a copper block frozen in liquid nitrogen for bottom‐up freeze casting. The frozen material was subsequently subjected to freeze‐drying, followed by heat treatment at 80 °C for 4 hours in an oven, resulting in the formation of the LCS aerogel (Figure 1b). The stirring process enhanced the strong interaction between hydrolyzed silane coupling agents and the –OH and –COOH groups in CNF‐C and LDH, while the freeze‐drying and heat‐treatment processes facilitated condensation reactions, resulting in the formation of a hydrophobic Si–O–Si binder on the surfaces of both CNF‐C and LDH (Figure 1c). The entire preparation process produced a cross‐linked network structure in which LDH acts as the rigid “brick,” CNF‐C functions as the flexible “mortar,” and Si–O–Si serves as the “binder.” Such “rigid‐flexible” architecture endows the LCS hybrid aerogel with both robust structural stability and hydrophobicity.

**Figure 2:**
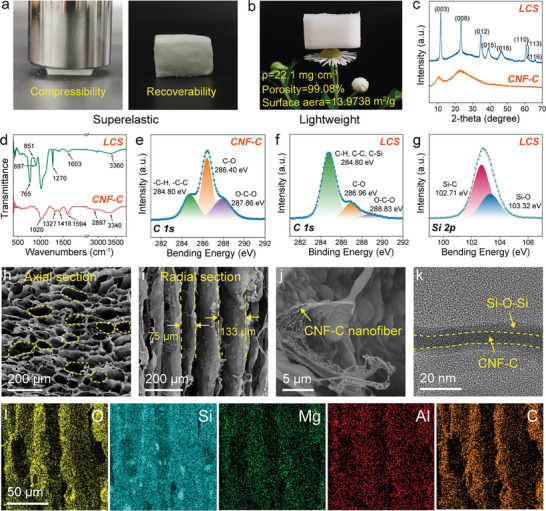
Microstructure of LCS hybrid aerogel. a, b) Digital photos of a) super‐elastic and b) ultralight LCS aerogel. c) XRD patterns. d) FT‐IR spectra. e) C 1s XPS spectrum of CNF‐C nanofibers. f, g) C 1s and Si 2p XPS spectra of LCS aerogel. h) Axial section, i) radial section, j) high magnification SEM images. k) TEM image and l) EDS mapping.

Driven by the simple preparation process and the unique building blocks, as‐prepared LCS hybrid aerogel demonstrates shape recovery even after being subjected to high‐strain compression (**Figure** **2**a). Additionally, LCS hybrid aerogel features an extremely low density (22.1 mg·cm^−3^) and high open porosity (99.08%), enabling it to remain stable on the flower's stamen without deformation (Figure 2b). This super‐elastic, lightweight aerogel is well‐suited for intricate application scenarios. To further elucidate the material's structural characteristics, X‐ray diffraction (XRD) analysis was performed on pure CNF‐C nanofibers and LCS hybrid aerogel, as illustrated in Figure 2c. For pure CNF‐C, a distinct (002) amorphous carbon diffraction peak appears at 22.85°. In contrast, the LCS aerogel exhibits additional sharp LDH diffraction peaks at 2θ = 11.56°, 23.33°, 34.66°, 39.24°, 46.47°, 60.74°, 62.03°, and 65.95°, corresponding to the (003), (006), (012), (015), (018), (110), (113), and (116) planes, respectively.^[^ ^12^ ^]^ These findings confirm the successful integration of highly crystalline LDH into the aerogel. In the Fourier transform infrared (FT‐IR) spectrum of pure CNF‐C (Figure 2d), absorption bands are observed at 3340, 2897, 1603, 1418, and 1020 cm^−1^, corresponding to the stretching vibrations of –OH, the tensile vibration of –CH_3_ and –CH_2_, the symmetric and asymmetric tensile vibrations –COOH, the stretching vibrations of –CH_2_, and the asymmetric stretching vibrations of C–O–C, respectively.^[^ ^13^ ^]^ For the LCS aerogel, absorption bands appear at 1603 cm^−1^ and 500–1010 cm^−1^, which are associated with the –OH stretching band of H_2_O and M–O lattice vibrations (M = Mg and Al) in LDH.^[^ ^14^ ^]^ Additionally, absorption bands at 1270 cm^−1^, 897 cm^−1^, and 765 cm^−1^ are attributed to the hydrophobic Si–O–Si binder, assigned respectively to the C–Si–O and O–Si–O units, the Si–OH unit, and the Si–CH_3_ unit, respectively. Furthermore, the FT‐IR spectrum of LCS hybrid aerogel shows significant changes compared to pure CNF‐C. The –COOH peak of CNF‐C shifts from 1594 cm^−1^ to 1603 cm^−1^, and the –OH peak at 3340 cm^−1^ migrates to 3360 cm^−1^. These shifts indicate intermolecular interactions between the building blocks, which contribute to the homogeneity and stability of the hybrid aerogel. X‐ray photoelectron spectroscopy (XPS) analysis was further conducted. The C 1s spectrum of CNF‐C can be deconvoluted into three peaks of C─H and C─C bonds at 284.8 eV, C─O bond at 286.96 eV, and O─C─O bond at 288.83 eV (Figure 2e). While the C 1s spectrum of LCS hybrid aerogel shows a significant increase in the area of the green peak at 284.8 eV due to the appearance of C–Si bonds (Figure 2f).^[^ ^15^ ^]^ As shown in Figure 2g, curve‐fitting of the Si 2p peaks reveals binding energies of 102.71 and 103.32 eV, which are consistent with Si─C and Si─O bonds, respectively.^[^ ^16^ ^]^ These findings collectively confirm the formation of Si–O–Si binder in LCS hybrid aerogel.

In this work, directional freeze‐drying was employed to precisely control ice crystal growth and regulate the microstructure of LCS hybrid aerogel to achieve specific performance targets. The axial cross‐section SEM image exhibits a tightly interconnected porous honeycomb structure, featuring large pores ranging from approximately 50–70 µm (Figure 2h). While the radial cross‐section displays a well‐organized layered morphology, with the size of the oriented channels ranging from approximately 75–133 µm (Figure 2i). The observed difference is attributed to the controlled directional freeze casting process. When the bottom of the mold was in contact with the frozen copper block, a bottom‐up temperature was established, thereby promoting ice crystal growth from the bottom upward and ultimately leading to the formation of parallel ice columns. Since the viscosity of LCS precursor solution was low, the movement of the cross‐linked network remained almost unrestricted, allowing it to be squeezed into the interstitial spaces as the ice crystals grew vertically. This process eventually resulted in the formation of an anisotropic pore structure after drying. As shown in Figure 2j, the cell walls are primarily composed of intertwined nanofibers. The nanopores formed by nanofiber winding (Figure S1, Supporting Information), together with the honeycomb‐like macropores, enrich the multistage pore structure of LCS aerogel, which can effectively reduce thermal conductivity. Transmission Electron Microscopy (TEM) analysis was performed to further investigate the microstructure. The image reveals an irregular structure with a diameter of approximately 1 µm. The edge structure suggests a layered configuration, which is likely the recrystallized LDH (Figure S2, Supporting Information). Additionally, the gray materials were found enveloping dark black nanofibers, as highlighted by the yellow arrows in Figure S3 (Supporting Information). Based on XPS results, it is speculated that the gray substance could be Si–O–Si, while the black material is likely CNF‐C (Figure 2k). Furthermore, the energy dispersive X‐ray spectroscopy (EDS) spectra reveal a uniform distribution of O, Si, Mg, Al, and C elements, confirming the evenly dispersed of the building blocks throughout LCS hybrid aerogel (Figure 2l and S4).

### 2.2 Compressible Mechanical Property

**Figure 3:**
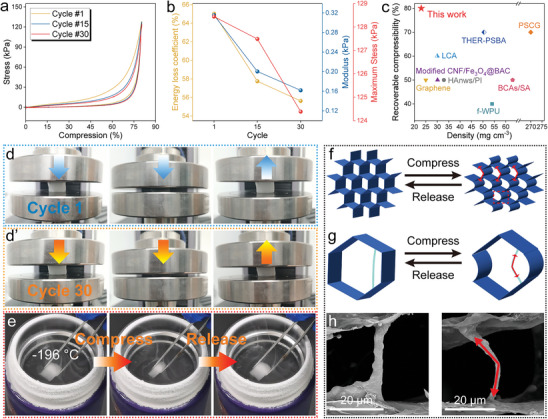
Mechanical properties of LCS hybrid aerogel. a) Radial cyclic compressive stress‐strain curves. b) Energy loss coefficient, maximum stress, and modulus of hybrid aerogel during cyclic compression test. c) Comparison of compressibility between LCS hybrid aerogel and other reported elastic aerogels. d) Digital photos during cyclic compression. e) Mechanical elasticity in liquid nitrogen. f, g) Elastic mechanism diagrams. h) SEM images.

Owing to the balanced rigid and flexible components in the “brick‐mortar‐binder” cross‐linked network of LCS hybrid aerogel, along with its specific microstructure, the LCS aerogel is expected to exhibit optimized mechanical performance. Figure S5 (Supporting Information) presents the stress‐strain curves for both radial and axial compression directions. In the initial stage, the stress shows a linear relationship with strain. At high strain levels (60%–80%), the pores in the aerogel are nearly closed, resulting in an increased density and a sharp rise in stress.^[^ ^17^ ^]^ Notably, due to the more ordered skeletal structure in the axial direction, the stress‐strain curve for axial compression demonstrates slightly higher compressive strength compared to the radial direction. While the aerogel is capable of fully rebounding in both compression directions, the axial energy loss coefficient is significantly higher than that of the radial direction, likely due to more concentrated stress transfer within the material. Consequently, radial compression is considered to exhibit better mechanical property. The radial cyclic compression curve shows that LCS aerogel exhibits stable elastic behavior (**Figure** **3**a). It can revert to its initial state without significant geometric deformation even after 30 cycles of 80% strain compression (Figure 3d), with a high stress retention rate of 96.84% (Figure 3b). Based on the slope of the stress‐strain curve within the range of 0% to 10%, the moduli of the aerogel are calculated to be 0.32 kPa, 0.20 kPa, and 0.16 kPa for the 1st, 15th, and 30th cycles, respectively. This reduction in modulus is attributed to the rearrangement or even fracture of the aerogel's network structure during deformation to accommodate compression. The decreasing energy loss coefficient with increasing cycle number further supports this observation.^[^ ^18^ ^]^ Additionally, compared with other elastic aerogels previously reported (Figure 3c),^[^ ^19^ ^]^ e.g., 50% for HAnws/PI aerogel with a density of 32.9 mg·cm^−3^,^[^ ^20^ ^]^ 70% for THER‐PSBA aerogel,^[^ ^21^ ^]^ and 50% for graphene aerogel with a density of 25 mg·cm^−3^,^[^ ^22^ ^]^ LCS aerogel possesses dual advantages of low density and high recoverable compressibility. Furthermore, the LCS hybrid aerogel exhibits remarkable mechanical robustness, as evidenced by its ability to compress and rapidly rebound to initial height for 15 cycles in ‐196 °C liquid nitrogen (Figure 3e; Figure S6, Movie S1, Supporting Information), demonstrating its potential for applications under extreme low‐temperature conditions.

The mechanical elasticity of LCS hybrid aerogel is primarily attributed to its unique structure and the synergistic enhancement effects of its components. As demonstrated in the schematic diagram (Figure 3f,g) and SEM images (Figure 3h), the cell walls and embedded fiber structures within the honeycomb pores will bend under external pressure. This bending process can generate effective support forces, enabling the LCS sample to rapidly return to its original shape upon the release of pressure.^[^ ^23^ ^]^ Additionally, the dense and robust “brick‐mortar‐binder” cross‐linked network effectively mitigates potential sliding or structural damage during compression^[^ ^24^ ^]^ Furthermore, molecular repulsion between the –CH_3_ groups in hydrophobic Si–O–Si binder not only prevents the collapse of LCS aerogel during preparation but also enhances its recovery performance.^[^ ^10^ ^]^ In contrast, hybrid aerogel without Si–O–Si component (LC aerogel) fails to rebound under applied pressure (Figure S7, Supporting Information). The outstanding mechanical elasticity and robustness of LCS hybrid aerogel will help reduce the risk of performance degradation or structural damage caused by external forces during use.

### 2.3 Hydrophobicity

**Figure 4:**
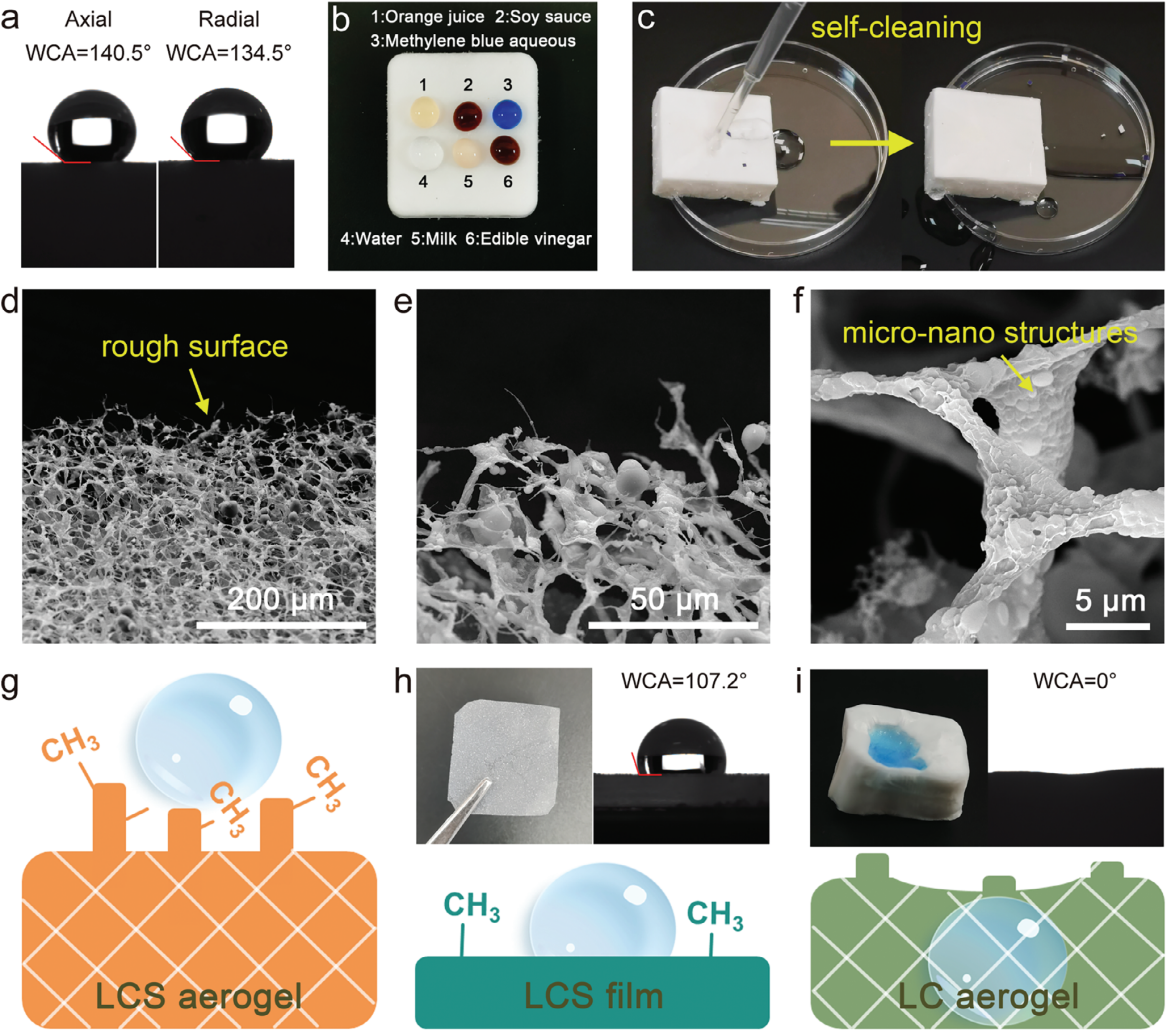
Hydrophobicity of LCS hybrid aerogel. a) WCAs. b) Wettability of aerogel against solvents. c) Self‐cleaning ability. d‐f) SEM images of the aerogel's surface. g–i) Wettability mechanism of g) LCS aerogel, h) LCS film, and i) LC aerogel.

The unique interfacial wetting property (hydrophobicity) of the LCS hybrid aerogel is crucial for its thermal insulation application. As shown in **Figure** **4**a, the water contact angles (WCAs) of the aerogel's lateral and cross‐sectional surfaces are 140.5° and 134.5°, respectively, demonstrating remarkable hydrophobicity. Additionally, the interior of the material also exhibits excellent hydrophobic properties (Figure S8, Supporting Information). This homogeneous hydrophobic performance is primarily attributed to the in‐situ formation of Si‐O‐Si, which effectively overcomes the depth limitations of hydrophobicity in traditional methods like chemical vapor deposition. Even if the surface layer is damaged, the newly exposed surface retains its hydrophobicity, significantly extending the aerogel's service life. Furthermore, Figure 4b demonstrates the lower wettability of LCS aerogel against various organic solvents, strongly indicating its potential as an anti‐fouling material. As illustrated in Figure 4c and Movie S2 (Supporting Information), solid residues adsorbed on the aerogel surface can be easily removed by water flow, demonstrating its self‐cleaning ability.

The wetting interface of LCS hybrid aerogel is attributed to the synergistic effect of its rough micro‐nano surfaces and low surface energy. As shown in Figure 4d,e, the high porosity and low‐density cross‐linking network endow the LCS hybrid aerogel with an extremely rough surface, promoting an increased volume of air retention below water droplets, thereby exhibiting non‐wettability to water.^[^ ^25^ ^]^ Figure 4f highlights the distinct micro‐nano structures on the skeleton. In addition, abundant –CH_3_ groups in hydrophobic Si–O–Si binder exposed on the micro‐nano surface (Figure 4g) create a strong steric hindrance, preventing water molecules from accessing high‐energy sites within the material and thus significantly reducing the water wettability.^[^ ^15^ ^]^ The results in Figure 4h,i further confirm this mechanism. Specifically, LC hybrid aerogel is completely hydrophilic, and LCS film without rough structure has limited hydrophobicity (107.24°). The excellent hydrophobic properties of LCS aerogel make it resistant to dirt and moisture during insulation, thereby extending its service life.

### 2.4 Thermal Insulation

**Figure 5:**
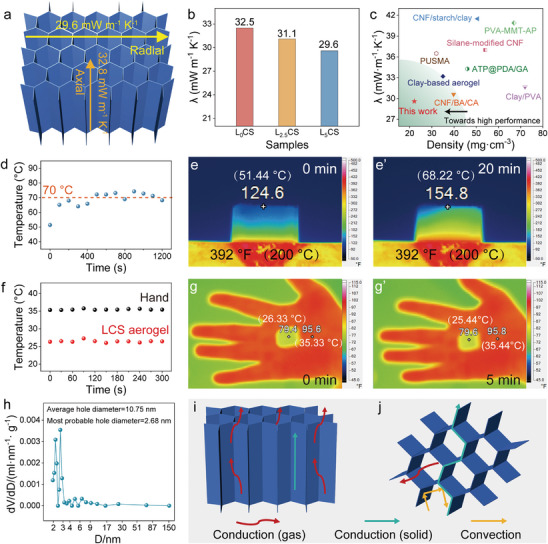
Thermal insulation performance of LCS aerogel. a) Thermal conductivities in different directions. b) Thermal conductivities of LCS hybrid aerogel with different LDH content. c) Comparison of thermal conductivities with other materials. d–g) Thermal insulation performance testing on d, e) hot sink and f, g) hand. h) Pore size curve. i, j) Thermal insulation mechanism.

Considering the low density (22.1 mg·cm^−3^), high porosity (>99%), good elasticity (80%), and excellent hydrophobicity (water contact angle = 140.49°) of LCS hybrid aerogel, it stands as a highly promising candidate for thermal insulation applications. The unique anisotropic microstructure of LCS aerogel significantly affects its thermal conductivity. Specifically, the radial thermal conductivity of the LCS hybrid aerogel is lower than its axial thermal conductivity (0.0296 W·m^−1^·K^−1^ vs 0.0328 W·m^−1^·K^−1^, **Figure** **5**a). Additionally, L_5_CS (LCS) aerogel displays the lowest thermal conductivity compared to both pure CNF‐C aerogel (0.0325 W·m^−1^·K^−1^) and L_2.5_CS aerogel (0.0311 W·m^−1^·K^−1^), highlighting the beneficial effect of LDH in reducing thermal conductivity (Figure 5b). Figure 5c provides a comparison of the thermal conductivities of recently published thermal management materials.^[^ ^26^ ^]^ Notably, our aerogel exhibits the lowest thermal conductivity among them. Additionally, considering the excellent mechanical elasticity of LCS aerogels, we gradually compressed the aerogel in the radial direction by 10%, 20%, and 30% to assess its impact on thermal conductivity. The results revealed that thermal conductivity increased with the rise in compressive strain (Figure S9, Supporting Information), confirming the potential to dynamically regulate the aerogel's thermal insulation performance by utilizing its compressive elasticity. This adaptability allows the aerogel to flexibly respond to varying temperatures and environmental conditions, such as automatically adjusting its insulation efficiency during seasonal changes or significant day‐night temperature fluctuations.

We further explored the practical application potential of LCS hybrid aerogel. The time‐dependent temperature curve (Figure 5d) and thermographic images (Figure 5e) demonstrate the thermal behavior of LCS hybrid aerogel when heated on a 200 °C hot sink for 20 min. The aerogel establishes thermal equilibrium with the surrounding environment within 400s, with the upper surface temperature stabilizing around 70 °C. After heating for 20 minutes, the upper surface temperature increased from the initial 51.44 °C to 68.22 °C, demonstrating the aerogel's outstanding thermal insulation capability. Moreover, when a 1.2 cm thick block of aerogel is placed directly on hand for 5 minutes, the upper surface temperature remains nearly constant throughout the test (Figure 5f) and the infrared radiation contrast between the hand covered by LCS aerogel and the background is too minimal to be detected by the infrared thermal imager (Figure 5g), indicating the potential of LCS aerogel in shielding infrared radiation.

Thermal conductivity is primarily composed of solid‐phase conduction (λ_s_), gas‐phase conduction (λ_g_), thermal convection (λ_c_), and thermal radiation (λ_r_). The anisotropic insulation mechanism of LCS hybrid aerogels is illustrated in Figure 5i,j. First, compared to axially aligned cell walls, the radially ordered honeycomb wall structure presents fewer channels for direct solid‐phase heat transfer. Additionally, the low density and high porosity result in a lower volume fraction of the solid phase, effectively reducing λ_s_. Second, the tubular pore channels enable heat flow to conduct upwards in the axial direction, whereas in the radial direction, heat flow tends to move laterally along the pore direction, thereby limiting radial λ_g_.^[^ ^27^ ^]^ Furthermore, the BET pore size distribution reveals the presence of a mesoporous structure in LCS aerogel wall (Figure 5h). Such a nanostructured composite aerogel wall hinders the free diffusion of gas molecules and further limits λ_g_.^[^ ^28^ ^]^ Third, the thin cell walls within the honeycomb structure enhance heat reflection and absorption attenuation, leading to a greater restriction of λ_c_ in the radial direction than in the axial direction. Moreover, the interpenetration of LDH within the aerogel confines air within the pores, contributing to the further reduction of λ_c_. Lastly, the interface between LDH and Si–O–Si, as well as the cross‐distribution of Mg and Al atoms, promotes phonon scattering. This interfacial thermal resistance results in heat loss at the interface during heat transfer.^[^ ^29^ ^]^ Additionally, the selective heat absorption by interlayer CO_3_^2−^ and the multilayer heat reflection by LDH laminates can effectively diminish λ_r_.^[^ ^4^ ^]^ In conclusion, LCS hybrid aerogel demonstrates outstanding thermal management performance. Given its remarkable hydrophobicity and exceptional mechanical stability in liquid nitrogen environments, LCS hybrid aerogel is a promising candidate for applications in building construction, transportation, and clothing.

### 2.5 Flame‐Retardancy

**Figure 6:**
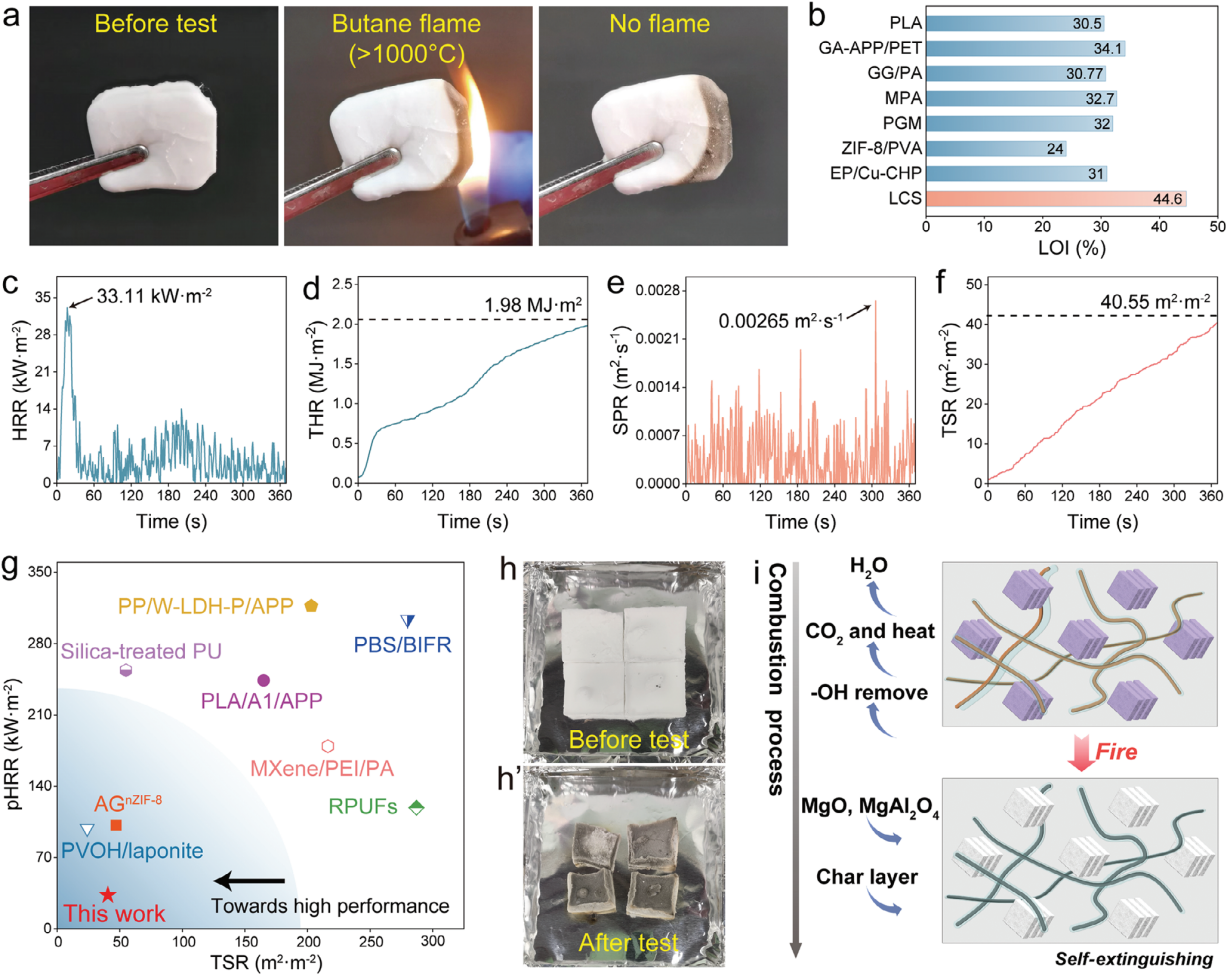
Flame‐retardancy of LCS hybrid aerogel. a) Optical pictures during ablation test. b) Comparison of LOI values with other materials. c‐f) HRR, THR, SPR and TSR curves. g) Comparison of pHRR and TSR values with other materials. h) Optical pictures of LCS aerogels before and after cone calorimetry test. i) Ablation resistance mechanism.

Thermal insulators with superior flame‐retardant performance can effectively reduce the risk of fire and explosion. Therefore, the ablation test, UL‐94 test, and LOI test were conducted to evaluate the flame‐retardancy of LCS aerogel.^[^ ^30^ ^]^ As illustrated in **Figure** **6**a and Movie S3 (Supporting Information), the ablation test demonstrates that no visible flame remained once the butane flame was removed. The LCS hybrid aerogel can reach V‐0 rating in the UL‐94 vertical burning test (Figure S10, Supporting Information), exhibiting excellent self‐extinguishing properties. Additionally, the LOI of the LCS hybrid aerogel was determined to be 44.6%, surpassing that of other reported flame‐retardant materials (Figure 6b), including PLA foam,^[^ ^31^ ^]^ GA‐APP/PET aerogel,^[^ ^32^ ^]^ GG/PA aerogel,^[^ ^33^ ^]^ MPA aerogel,^[^ ^34^ ^]^ PGM aerogel,^[^ ^35^ ^]^ ZIF‐8/PVA aerogel,^[^ ^36^ ^]^ and EP/Cu‐CHP composite.^[^ ^37^ ^]^ To further analyze the thermal behavior of LCS aerogel during the fire development stage, a cone calorimetric test was conducted. As expected, the LCS hybrid aerogel remained non‐ignitable during the test. Upon exposure to thermal radiation, the heat release rate (HRR) of the sample reaches a peak of 33.11 kW·m^−2^ after a certain period and then stabilized (Figure 6c), with a total heat release (THR) of 1.98 MJ·m^2^ (Figure 6d). The smoke production rate (SPR) of the aerogel remained relatively steady, with a maximum value of 0.00265 m^2^·s^−1^ (Figure 6e). The total smoke release (TSR) is 40.55 m^2^·m^−2^ (Figure 6f), which is only 1/5–1/10 of that produced by commercially available polymer foam (200 to 400 m^2^·m^−2^).^[^ ^38^ ^]^ Figure 6g summarizes the peak heat release rate (pHRR) and TSR of the LCS hybrid aerogel and other representative flame‐retardant materials.^[^ ^39^ ^]^ The results indicate that our aerogel exhibits the best flame‐retardant performance. Figure 6h shows optical images of the aerogel before and after the cone calorimeter test. The LCS hybrid aerogel underwent a color change from white to black, which is attributed to the solid‐state oxidation in the absence of flame. This result is consistent with the smoldering phenomenon observed in the butane flame test.

The excellent flame‐retardant performance of LCS hybrid aerogel relies on the synergistic effect of multiple mechanisms (Figure 6i). During heating, bound H_2_O, ‐OH groups, and interlayer CO_3_^2−^ are gradually released in the form of H_2_O and CO_2_, accompanied by a significant endothermic process, which effectively reduces the combustion temperature. As H_2_O, CO_2_, and –OH are released at distinct temperatures (207 °C, 291 °C, and 416 °C), LCS aerogel can provide continuous fire protection across a broad temperature range.^[^ ^40^ ^]^ Additionally, the released H_2_O and CO_2_ can dilute and block combustible gases, thus weakening the conditions for combustion.^[^ ^41^ ^]^ Furthermore, the solid products (MgO and MgAl_2_O_4_, Figure S11, Supporting Information) generated by LDH are beneficial to the formation of char layer produced by CNF‐C, forming a protective barrier on the sample's surface. This layer effectively prevents oxygen infiltration, slows down combustion, and suppresses smoke generation.^[^ ^42^ ^]^ Most importantly, the aerogel's unique structural characteristics, including its extremely high porosity and low thermal conductivity, significantly impede heat transfer, further enhancing its flame‐retardant property.

## 3 Conclusion

To summarize, we have developed an LCS hybrid aerogel with a distinctive “brick‐and‐mortar” network. Its oriented pore structure, the synergistic effects between rigid and flexible building blocks, along with the molecular repulsion effect of –CH_3_ groups, result in excellent mechanical elasticity and robustness under repeated compression at extremely low temperature. In addition, with high surface roughness and low surface energy, LCS hybrid aerogel demonstrates good hydrophobic properties. The material also exhibits excellent flame‐retardant performance due to its endothermic decomposition, dilution and barrier, and protective film formation effects during combustion. Furthermore, the tailored structure resulting from directional freeze casting endows the aerogel with excellent thermal management and infrared shielding capacities. The successful fabrication of this LCS hybrid aerogel offers new insights into designing thermal management materials with high adaptability for extreme conditions.

## 4 Experimental Section

### Materials

Mg/Al layered double hydroxide (Mg/Al LDH) was purchased from Kisuma Chemicals. Carboxylated cellulose nanofibers (CNF‐C) aqueous dispersion (1 wt%, carboxyl content 1.2‐3.0 mmol·g^−1^) was purchased from Guilin Qihong Technology Co., Ltd (China). Methyltrimethoxysilane (MTMS) and Vinyltriethoxysilane (VTES) were brought from Shanghai Macklin Biochemical Technology Co., Ltd (China). CH_3_COOH was obtained from Aladdin Reagent Co., Ltd (China). All chemicals were purchased from reagent manufacturers and used without any additional purification.

### Preparation of LCS Hybrid Aerogel

Firstly, the LDH was heat‐treated at 450 °C for 600 min. The resulting material was then dispersed in water and magnetically stirred at 600 r·min^−1^ at 80 °C for 4 h to form a uniform LDH dispersion (20 mg·mL^−1^). Subsequently, 1 mL MTMS and 50 µL VTES were added to 3 mL of CH_3_COOH solution (pH = 4), and the mixture was stirred for 2 hours to undergo hydrolysis and form silanol. Next, 800 µl of silanol and 0.25 mL of LDH (5 mg) dispersion were sequentially added to 5 g of 0.3 wt% CNF‐C suspension and stirred at room temperature for 1 h to form a homogeneous precursor suspension. The appropriate amount of precursor suspension was then placed in a mold with its bottom in contact with the top of a copper block, which was cooled using liquid nitrogen. The frozen material was then freeze‐dried for 72 h. Finally, the obtained aerogel was heat‐treated in an oven at 80 °C for 4 h, yielding the final LCS hybrid aerogel.

The resulting aerogel can also be referred to as L_5_CS hybrid aerogel, where 5 indicates that the mass of LDH in the prepared aerogel was 5 mg. The mass of LDH in the L_2.5_CS hybrid aerogel was 2.5 mg. In the L_0_CS aerogel, the mass of LDH was 0 mg. The LCS film was obtained by directly drying the precursor suspension in an oven at 80 °C for 4 h. The LC hybrid aerogel was produced by freeze‐drying and heat‐treating the CNF‐C and LDH suspension without silanol.

### Characterization

Scanning electron microscopy (SEM, TESCAN) and energy dispersive X‐ray spectrometer (EDS) were used to characterize the morphologies and chemical element distribution of LCS, respectively. The X'Pert PRO diffractometer with Cu Kα radiation (λ = 1.5178 Å, 40 kV × 40 mA) was used to obtain the X‐ray diffraction (XRD) patterns. Fourier transform infrared (FT‐IR) spectroscopy (PerkinElmer) and X‐ray photoelectron spectroscopy (XPS) were employed to characterize the chemical composition of the sample surfaces. The automatic true density analyzer (TD1‐1539) was used to obtain the open porosity. The specific surface area and pore‐size distribution were determined by nitrogen adsorption‐desorption method using a Brunauer‐Emmett‐Teller (BET) Analyzer (Micromeritics ASAP 2460). The limiting oxygen index (LOI) was carried out using FTT0077. The vertical burning test (UL‐94) was performed according to GB/T 2408–2008, and the dimensions of all samples were 150 mm × 12 mm × 7mm.

### Mechanical Elasticity

The samples were tested on a SUNS‐UTM 2203 with a speed of 10.0 mm min^−1^ and a prestress of 0.05 N. In addition, LCS aerogels were tested radially to evaluate mechanical properties. In addition, cyclic elastic tests were conducted on the rectangular sample at 80% strain (30 cycles).

### Hydrophobicity

The contact angle meter (JC2000D1) was used to test the wettability of water droplets on the sample surface. Orange juice, soy sauce, methylene blue aqueous, water, milk, and edible vinegar were placed on a square aerogel with a length of 32 mm and a width of 30 mm to evaluate its hydrophobicity. To assess the water adhesion force and self‐cleaning capability, the aerogel was inclined at an angle of less than 10°, and the self‐cleaning ability was confirmed by observing whether rolling water droplets could effectively remove impurities from the surface.

### Thermal Insulation

LCS hybrid aerogel with a size of 21.5 mm × 17.8 mm × 12 mm was placed on the platform with the lower surface subjected to continuous heating at a constant temperature of 200 °C. The time‐dependent upper surface temperature of aerogels upon heating was investigated.

### Cone Calorimetry

As 700 °C generally indicates the transition from the initial stage to the development stage of the solid fire, a thermal radiation intensity of 35 kW·m^−2^ (713 °C) was chosen for oxygen‐consumption cone calorimetry test of LCS hybrid aerogel to simulate the fire development stage. A forced combustion test was performed in an oxygen‐consumption cone calorimeter (VOUCH 6810) using square samples.

## Conflict of Interest

The authors declare no conflict of interest.

## Supporting information



Supporting Information

Supplemental Movie 1

Supplemental Movie 2

Supplemental Movie 3

## Data Availability

The data that support the findings of this study are available from the corresponding author upon reasonable request.
