# Supplementary material for: “Brick‐Mortar‐Binder” Design toward Highly Elastic, Hydrophobic, and Flame‐Retardant Thermal Insulator
Source: Adv Sci (Weinh). 2024 Nov 29;12(4):2410938. doi: 10.1002/advs.202410938 (PMC11775557; doi:10.1002/advs.202410938)
Supplement: Supplementary file 1 — Supporting Information [file ADVS-12-2410938-s001.docx]

Supporting Information

“Brick-mortar-binder” Design Toward Highly Elastic, Hydrophobic, and Flame-retardant Thermal Insulator

*Shanying Sui, Huafeng Quan,* Jingxing Wang, Yufang Lu, Yufan Yang, Yuhan Sheng, Zhifangs Sun, and Yi Zhang**


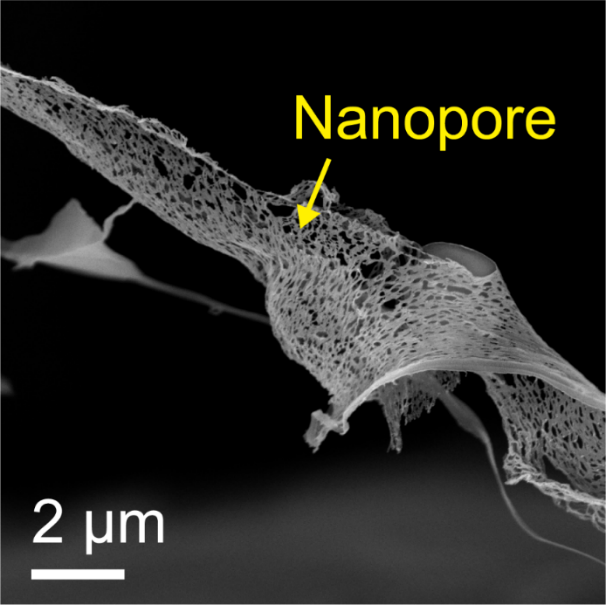


**Figure S1.** High magnification SEM images of LCS hybrid aerogel.


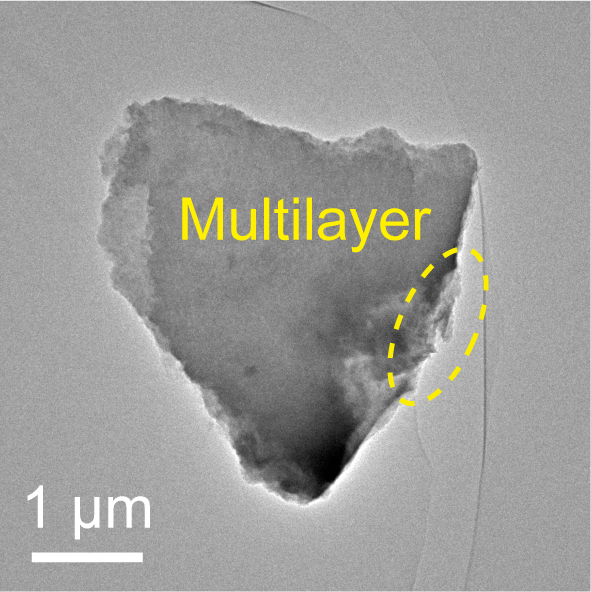


**Figure S2.** TEM image of LCS hybrid aerogel.


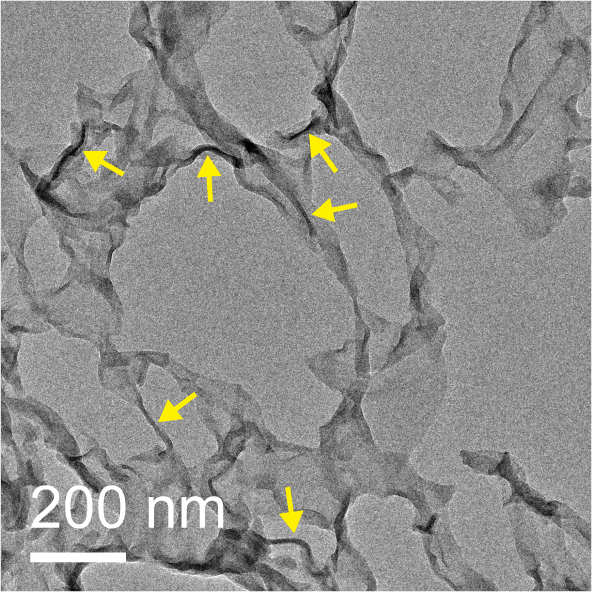


**Figure S3.** TEM image of LCS hybrid aerogel.


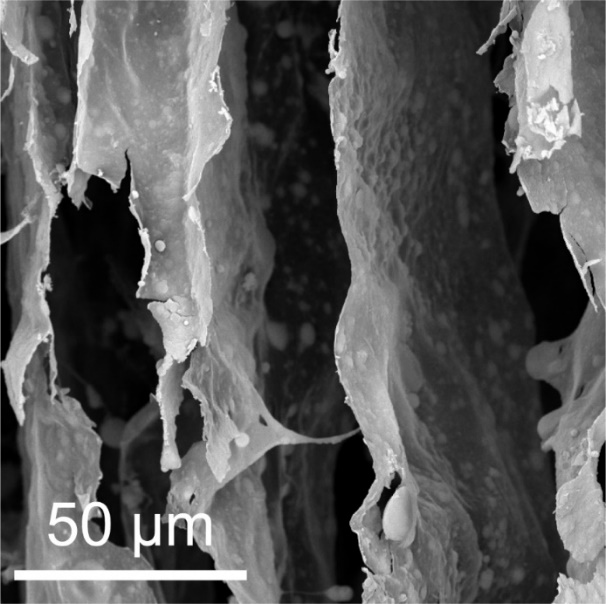


**Figure S4.** SEM image of LCS hybrid aerogel.


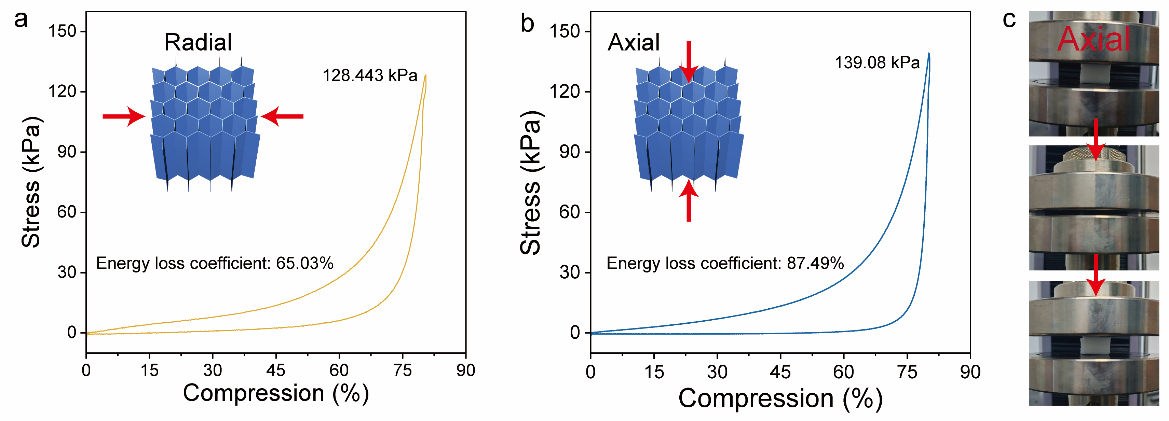


**Figure S5.** a) Radial compressive stress-strain curve. b) Axial compressive stress-strain curve. c) Digital photos of LCS hybrid aerogel during compression.


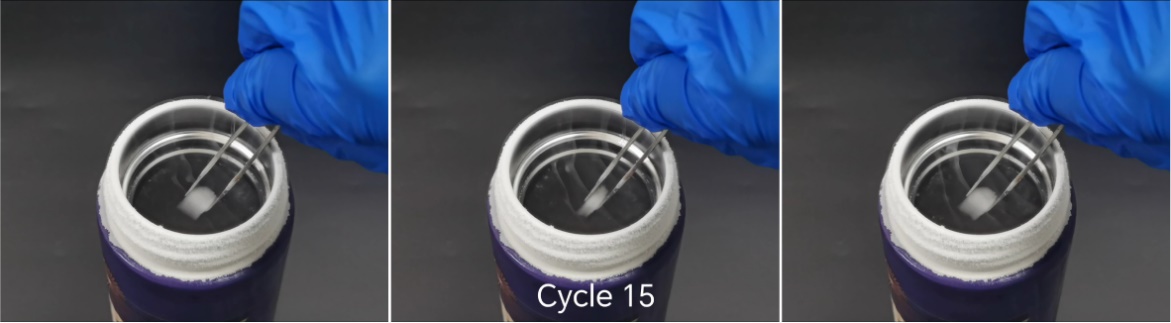


**Figure S6.** Mechanical elasticity of LCS hybrid aerogel after 15 cycles of compression under liquid nitrogen.


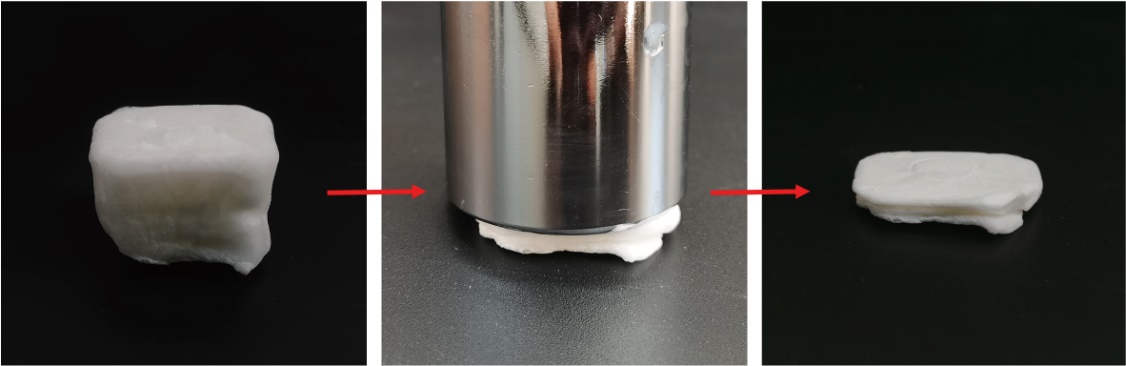


**Figure S7.** Mechanical elasticity of hybrid aerogel without Si-O-Si (LC aerogel).


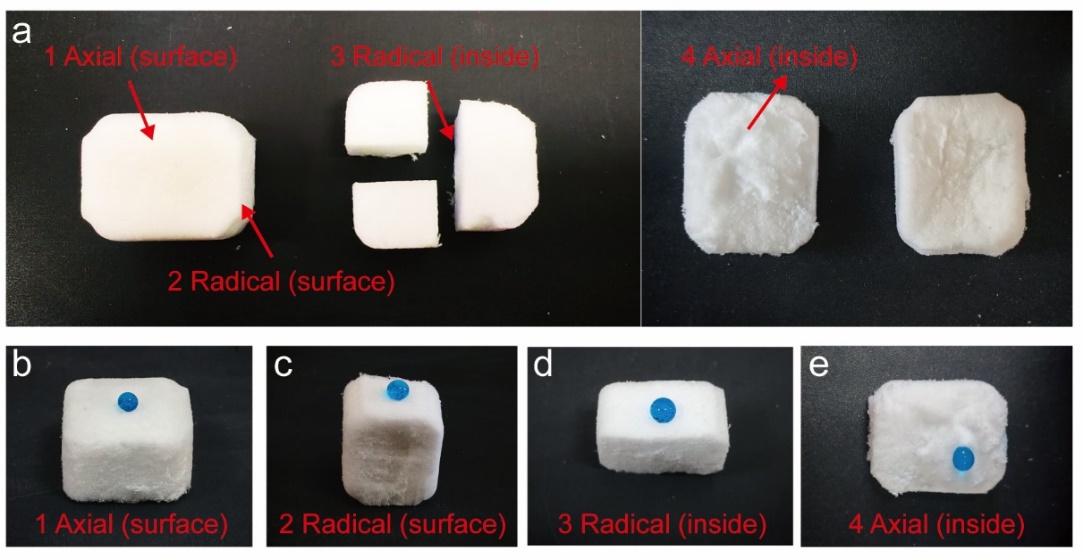


**Figure S8.** a) Schematic diagram of hydrophobic test direction of LCS aerogel. b-e) Hydrophobic properties of LCS aerogel in various test directions.


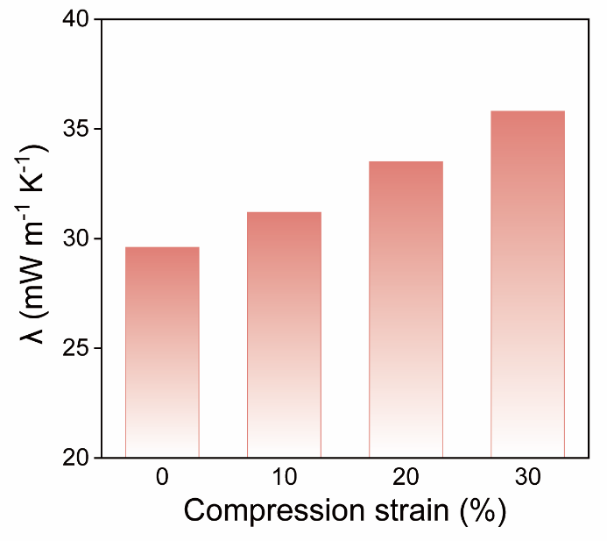


**Figure S9.** Thermal conductivities of LCS aerogel under different radial compressive strains.


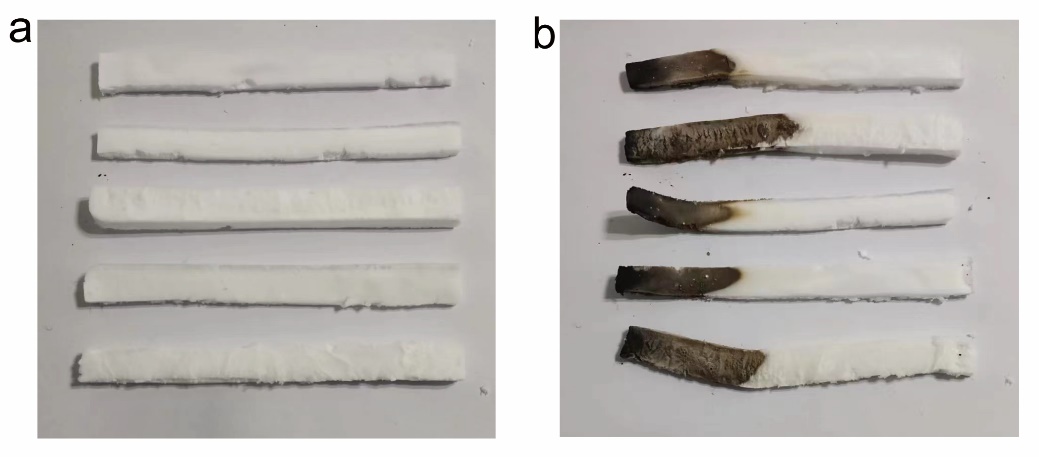


**Figure S10.** Digital photos of aerogels a) before and b) after UL-94 test.


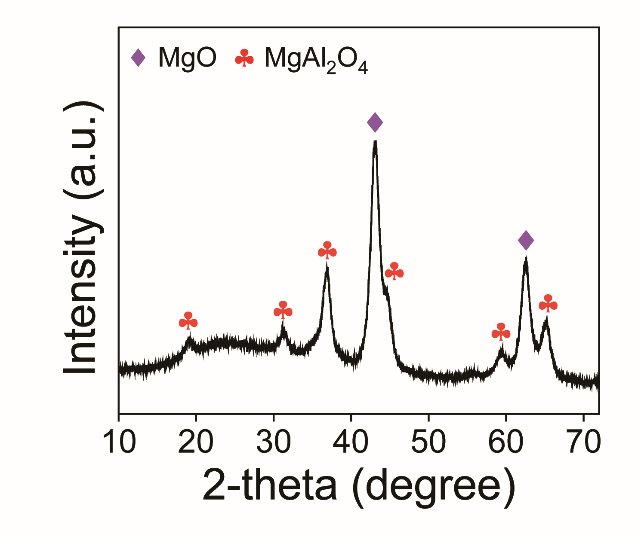


**Figure S11.** XRD pattern of recrystallized LDH in LCS aerogel after heat treatment at 850 ℃.

**Movie S1** Mechanical elasticity of LCS hybrid aerogel after 15 cycles of compression under liquid nitrogen.

**Movie S2** Self-cleaning performance of LCS hybrid aerogel.

**Movie S3** Flame-retardancy of LCS hybrid aerogel.
